# Polymeric Plant-derived Excipients in Drug Delivery

**DOI:** 10.3390/molecules14072602

**Published:** 2009-07-16

**Authors:** Carien E. Beneke, Alvaro M. Viljoen, Josias H. Hamman

**Affiliations:** Department of Pharmaceutical Sciences, Tshwane University of Technology, Private Bag X680, Pretoria, 0001, South Africa; E-mails: cegpharm@live.com (C-E.B.); viljoenam@tut.ac.za (A-M.V.)

**Keywords:** polysaccharide, polymer, excipient, drug delivery, controlled release, renewable resource

## Abstract

Drug dosage forms contain many components in addition to the active pharmaceutical ingredient(s) to assist in the manufacturing process as well as to optimise drug delivery. Due to advances in drug delivery technology, excipients are currently included in novel dosage forms to fulfil specific functions and in some cases they directly or indirectly influence the extent and/or rate of drug release and absorption. Since plant polysaccharides comply with many requirements expected of pharmaceutical excipients such as non-toxicity, stability, availability and renewability they are extensively investigated for use in the development of solid oral dosage forms. Furthermore, polysaccharides with varying physicochemical properties can be extracted from plants at relatively low cost and can be chemically modified to suit specific needs. As an example, many polysaccharide-rich plant materials are successfully used as matrix formers in modified release dosage forms. Some natural polysaccharides have even shown environmental-responsive gelation characteristics with the potential to control drug release according to specific therapeutic needs. This review discusses some of the most important plant-derived polymeric compounds that are used or investigated as excipients in drug delivery systems.

## 1. Introduction

Polymers have been successfully employed in the formulation of solid, liquid and semi-solid dosage forms and are specifically useful in the design of modified release drug delivery systems. Both synthetic and natural polymers have been investigated extensively for this purpose [1,2], but the use of natural polymers for pharmaceutical applications is attractive because they are economical, readily available, non-toxic, capable of chemical modifications, potentially biodegradable and with few exceptions, also biocompatible [3,4,5,6]. Of increasing importance is the fact that plant resources are renewable and if cultivated or harvested in a sustainable manner, they can provide a constant supply of raw material [7]. However, substances from plant origin also pose several potential challenges such as being synthesised in small quantities and in mixtures that are structurally complex, which may differ according to the location of the plants as well as other variables such as the season. This may result in a slow and expensive isolation and purification process. Another issue that has become increasingly important is that of intellectual property rights [8,9].

Traditionally, excipients were included in drug formulations as inert vehicles that provided the necessary weight, consistency and volume for the correct administration of the active ingredient, but in modern pharmaceutical dosage forms they often fulfil multi-functional roles such as improvement of the stability, release and bioavailability of the active ingredient, enhancement of patient acceptability and performance of technological functions that ensure ease of manufacture [10].

The specific application of plant-derived polymers in pharmaceutical formulations include their use in the manufacture of solid monolithic matrix systems, implants, films, beads, microparticles, nanoparticles, inhalable and injectable systems as well as viscous liquid formulations [11,12,13]. Within these dosage forms, polymeric materials have fulfilled different roles such as binders, matrix formers or drug release modifiers, film coating formers, thickeners or viscosity enhancers, stabilisers, disintegrants, solubilisers, emulsifiers, suspending agents, gelling agents and bioadhesives [1]. 

Polymers are often utilised in the design of novel drug delivery systems such as those that target delivery of the drug to a specific region in the gastrointestinal tract or in response to external stimuli to release the drug. This can be done via different mechanisms including coating of tablets with polymers having pH dependent solubilities or incorporating non-digestible polymers that are degraded by bacterial enzymes in the colon. Non-starch, linear polysaccharides are resistant to the digestive action of the gastrointestinal enzymes and retain their integrity in the upper gastrointestinal tract. Matrices manufactured from these polysaccharides therefore remain intact in the stomach and the small intestine, but once they reach the colon they are degraded by the bacterial polysaccharidases. This property makes these polysaccharides exceptionally suitable for the formulation of colon-targeted drug delivery systems [4,14]. 

This review discusses the use of plant-derived polymers and their semi-synthetic derivatives as excipients in the formulation of drug delivery systems. Specific reference is made to the use of natural polymers in the design of novel dosage forms such as modified release matrix type tablets and other new drug delivery systems under investigation. 

## 2. Cellulose

The polysaccharides of the plant cell wall consist mainly of cellulose, hemicelluloses and pectin [15]. Cellulose is an essential structural component of cell walls in higher plants and is the most abundant organic polymer on earth. This linear, unbranched polysaccharide consists of β-1,4-linked d-glucose units and many parallel cellulose molecules form crystalline microfibrils that are mechanically strong and highly resistant to enzymatic attack. These long crystalline ribbons are aligned with each other to provide structure to the cell wall. Cellulose is insoluble in water and indigestible by the human body [16,17]. 

**Figure 1:**
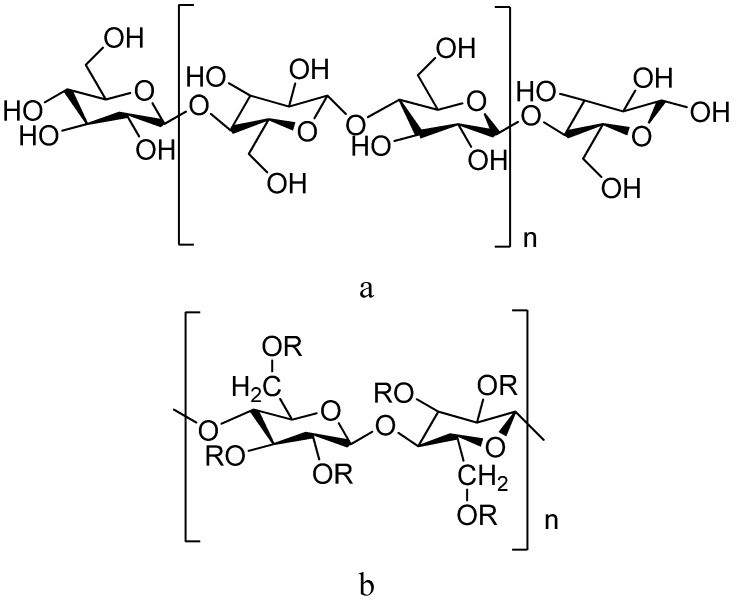
Chemical structure of a) powdered cellulose (n ≈ 500) or microcrystalline cellulose (n ≈ 220) and b) hydroxypropylmethylcellulose.

Powdered cellulose (whose chemical structure is shown in Figure 1) is mechanically disintegrated cellulose obtained as a pulp from fibrous materials such as wood or cotton and although it was used in pharmaceutical applications such as a filler in tablets, it is microcrystalline cellulose that represents a novel and more useful cellulose powder. Microcrystalline cellulose is partially depolymerised cellulose prepared by treating high quality cellulose with hydrochloric acid to produce free flowing non-fibrous particles. Microcrystalline cellulose is mainly used in the pharmaceutical industry as a diluent/binder in tablets for both the granulation and direct compression processes [18,19]. 

The hydroxyl moieties on the d-glucopyranose units of the cellulose polymer offer a variety of possibilities for the formation of derivatives. Cellulose derivatives can be made by means of etherification (e.g. hydroxypropylmethylcellulose, carboxymethylcellulose), esterification (cellulose nitrate, cellulose acetate, cellulose acetate phthalate), cross-linking or graft copolymerisation [18]. Controlled release applications for cellulose derivatives include the formulation of membrane controlled drug release systems or monolithic matrix systems. Film coating techniques for the manufacture of membrane controlled release systems include enteric coated dosage forms and the use of semi-permeable membranes in osmotic pump delivery systems. Cellulose acetate was amongst the first materials used to prepare semi-permeable membranes for osmotic pump dosage forms and cellulose esters as well as cellulose ester blends are continually used commercially for this purpose [20]. However, monolithic matrix systems are amongst the most popular technologies for controlled drug delivery because of their simplicity of formulation, ease of manufacture, low cost, acceptance and applicability to drugs with a wide range of solubility [21,22]. 

Cellulose ethers are prepared by replacing the hydroxyl groups with either alkyl or hydroxylalkyl groups [18]. Hydroxypropylmethylcellulose (chemical structure is shown in Figure 1) is a partly *O*-methylated and *O*-(2-hydroxypropylated) cellulose ether derivative that has been extensively investigated as an excipient in controlled release drug delivery systems due to its gel forming ability [1,23]. In a study where two cellulose ethers; hydroxypropylmethylcellulose and carboxymethyl-cellulose were employed as polymeric carrier materials in matrix tablets for controlled release of a soluble drug, diltiazem, it was found that each polymer on its own could sustain drug release over an extended period of time in these systems. More importantly, a mixture of the two cellulose ethers in the matrix type tablets enabled zero order drug release kinetics at both pH 4.5 and 6.8 [24]. Hydroxypropylmethylcellulose monolithic matrix systems showed similar dissolution profiles as a commercial osmotic pump system for glipizide, a drug with low solubility. It was further found that the hydroxypropylmethylcellulose matrix systems have a stronger gel structure than those made of polyethylene oxide, which may provide superior *in vivo* performance in terms of matrix resistance to the destructive forces within the gastrointestinal tract [22].

where R is H, CH_3_ or [CH_3_CH(OH)CH_2_].

## 3. Hemicellulose

Hemicellulose consists of a group of complex polysaccharides that are bound to the surface of cellulose microfibrils but their structure prevents them from forming microfibrils by themselves. Hemicellulose polysaccharides consist of xyloglucans, xylans and mannans that can be extracted from the plant cell wall with a strong alkali. They have backbones made up of β-1,4-linked d-glycans. Xyloglucan has a similar backbone as cellulose, but contains xylose branches on 3 out of every 4 glucose monomers. The β-1,4-linked d-xylan backbone of arabinoxylan contains arabinose branches [16,17,25,26]. 

Glucomannan is a hydrocolloidal polysaccharide of the mannan family consisting of β-1,4 linked d-mannose and d-glucose monomers (with acetyl side branches on some of the backbone units), but the mannose:glucose ratio may differ depending on the source. The acetyl groups contribute to its solubility and swelling capacity and assist in making it a soluble natural polysaccharide with the highest viscosity and water-holding capacity. It is very abundant in Nature and this polysaccharide is specifically derived from softwoods, roots, tubers and plant bulbs. The most commonly used type of glucomannan is referred to as konjac glucomannan, which is extracted from the tubers of *Amorphophallus konjac* K. Koch and is a very promising polysaccharide for incorporation into drug delivery systems. Since konjac glucomannan by itself forms very weak gels, it has been investigated as an effective excipient in controlled release drug delivery devices in combination with other polymers or by modifying its chemical structure [13,27]. 

It was shown that konjac glucomannan gel systems were able to maintain integrity and control the release of theophylline and diltiazem for 8 hours. This was, however, dependent on the country of origin (i.e. Japan, Europe or America) due to differences in the degree of acetylation of the konjac glucomannan [27]. Matrix tablets prepared from konjac glucomannan alone showed the ability to sustain the release of cimetidine in the physiological environments of the stomach and small intestines but the presence of β-mannanase (colon) accelerated the drug release substantially. Mixtures of konjac glucomannan and xanthan gum in matrix type tablets showed high potential to sustain and control the release of the drug due to stabilisation of the gel phase of the tablets by a network of intermolecular hydrogen bonds between the two polymers to effectively retard drug diffusion [28]. Wen *et al.,* 2008 [29] used konjac glucomannan to form hydrophilic cylinders and particles for controlled release of DNA. Konjac glucomannan cross-linked with trisodium trimetaphosphate formed hydrogel systems that could sustain hydrocortisone release dependent on cross-linking density and enzymatic degradation [30].

## 4. Pectin

**Figure 2:**
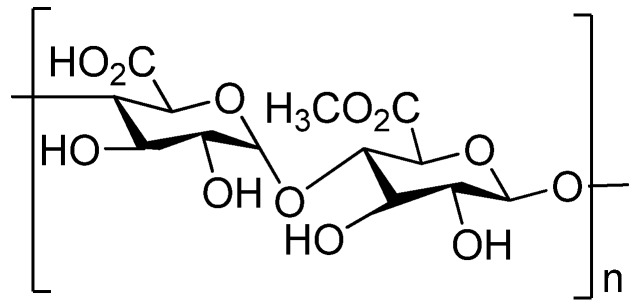
Chemical structure of pectin.

Pectin (chemical structure is shown in Figure 2) is a family of complex polysaccharides present in the walls that surround growing and dividing plant cells. It is also present in the junctional zone between cells within secondary cell walls including xylem and fiber cells in woody tissue [31,32]. Pectin is an essential component in the initial growth and ripening process of fruit and is often a waste material from the food and fruit processing industry with a consequent high availability [33]. The main component of pectin is a linear polysaccharide composed of α-1,4-linked d-galacturonic acid units, but the linear structure is interrupted with highly branched regions. The galacturonic acid polysaccharides are rich in neutral sugars such as rhamnose, arabinose, galactose, xylose and glucose. The composition of pectin can vary based on the botanical source, for example pectin from citrus contains less neutral sugars and has a smaller molecular size compared to pectin obtained from apples [34,35,36]. 

Pectin has been investigated as an excipient in many different types of dosage forms such as film coating of colon-specific drug delivery systems when mixed with ethyl cellulose, microparticulate delivery systems for ophthalmic preparations and matrix type transdermal patches. It has high potential as a hydrophilic polymeric material for controlled release matrix drug delivery systems, but its aqueous solubility contributes to premature and fast release of the drug from these matrices [14]. 

One of the options to reduce the high solubility of pectin in aqueous medium is through chemical modification without affecting favourable biodegradability properties. Pectins can be chemically modified by saponification catalysed by mineral acids, bases, salts of weak acids, enzymes, concentrated ammonium systems and primary aliphatic amines. Calcium salts of pectin have reduced solubility and matrix tablets prepared with calcium pectinate showed very good potential to be used in colon-targeted drug delivery systems. Furthermore, cross-linking of pectin with calcium ions inhibits the release of the incorporated drug from pectin tablets by suppressing both the dissolution and swelling of these systems [33,37,38,39]. 

Depending on the type and structure of the pectin molecule, pectins can gel in various ways. Gelling can be induced by acid or cross-linking with calcium ion or by reaction with alginate. When a pectin solution is titrated with acid, the ionization of carboxylate groups on pectins is repressed causing pectin molecules to no longer repel each other over their entire chains. The pectins can thus associate over a portion of their chains to form acid-pectin gels. Gel forming systems have been investigated widely for sustained drug delivery. A mixture of xyloglucan with pectin resulted in an *in situ* gel forming system with sustained paracetamol drug delivery in rats [38,40]. 

## 5. Inulin

**Figure 3:**
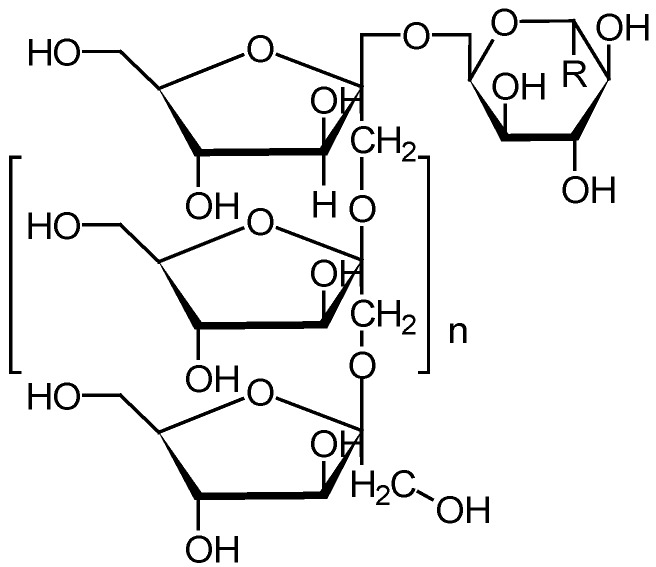
Chemical structure of inulin.

Inulin (chemical structure is shown in Figure 3) consists of a mixture of oligomers and polymers that belong to the group of gluco-fructans and occur in plants such as garlic, onion, artichoke and chicory. The inulin molecules contain from two to more than 60 fructose molecules linked by β-2,1-bonds. Inulin is resistant to digestion in the upper gastrointestinal tract, but is degraded by colonic microflora [41,42]. 

Inulin with a high degree of polymerisation was used to prepare biodegradable colon-specific films in combination with Eudragit^®^ RS that could withstand break down by the gastric and intestinal fluids [41]. It was shown in another study where different Eudragits^®^ were formulated into films with inulin that when a combination of Eudragit^®^ RS and Eudragit^®^ RL was mixed with inulin it exhibited better swelling and permeation properties in colonic medium rather than other gastrointestinal media [43]. Methylated inulin hydrogels were developed as colon-specific drug delivery systems and investigated for water uptake and swelling. The hydrogels exhibited a relatively high rate of water uptake and anomalous dynamic swelling behaviour [42]. 

Inulin derivatised with methacrylic anhydride and succinic anhydride produced a pH sensitive hydrogel by UV irradiation that exhibited a reduced swelling and low chemical degradation in acidic medium, but it had a good swelling and degradation in simulated intestinal fluid in the presence of its specific enzyme, inulinase [44].

## 6. Alginates

**Figure 4:**
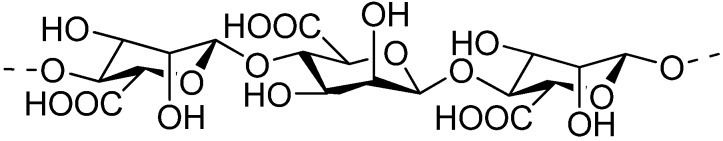
Chemical structure of alginates.

Alginates or alginic acids (chemical structure is shown in Figure 4) are linear, unbranched polysaccharides found in brown seaweed and marine algae such as *Laminaria hyperborea*, *Ascophyllum nodosum* and *Macrocystis pyrifera*. These polymers consist of two different monomers in varying proportions, namely β-d-mannuronic acid and α-l-guluronic acid linked in α- or β-1,4 glycosidic bonds as blocks of only β-d-mannuronic acid or α-l-guluronic acid in homopolymers or alternating the two in heteropolymeric blocks. Alginates have high molecular weights of 20 to 600 kDa [16,45,46]. 

Alginates have been used and investigated as stabilizers in emulsions, suspending agents, tablet binders and tablet disintegrants [47]. The gelling properties of alginate’s guluronic residues with polyvalent ions such as calcium or aluminium allow cross-linking with subsequent formation of gels that can be employed to prepare matrices, films, beads, pellets, microparticles and nanoparticles [48,49]*.* Dual drug-loaded alginate beads containing drug in inner and outer layers were prepared by dropping single-layered alginate beads into a CaCl_2_ solution and their drug release characteristics were investigated in simulated gastric fluid followed by intestinal fluid. The beads protected the drug in the gastric fluid with no release of the drug, while a biphasic release (i.e. a linear release for the first four hours and another linear phase thereafter) was obtained when the dissolution medium was changed to intestinal fluid [50]. 

The *in vivo* delivery of anti-tuberculosis drugs was investigated in mice for alginate nanoparticles prepared by cation induced gelation. A single oral dose achieved therapeutic drug concentrations in the blood plasma for 7-11 days and in organs such as the lungs, liver and spleen for a total of 15 days. The drugs encapsulated into these nanoparticles resulted in significantly higher bioavailability compared to the free drug. Furthermore, in *M. tuberculosis* infected mice only three oral doses of the nanoparticles that were spaced 15 days apart resulted in complete bacterial clearance from specific organs, which is comparable to 45 conventional doses of the free drug [51].

In a study where different grades of sodium alginate with different particle size distributions, viscosities and chemical compositions were used as drug release modifiers, it was found that the mentioned variables influence swelling, erosion and drug release from matrix type tablets, especially in a neutral environment. It was shown that sodium alginate matrix type tablets are able to sustain drug release even for highly soluble drug candidates. Furthermore, the drug release data obtained in a neutral dissolution medium correlated well with a zero-order kinetics model [35,45].

## 7. Carrageenans

**Figure 5:**
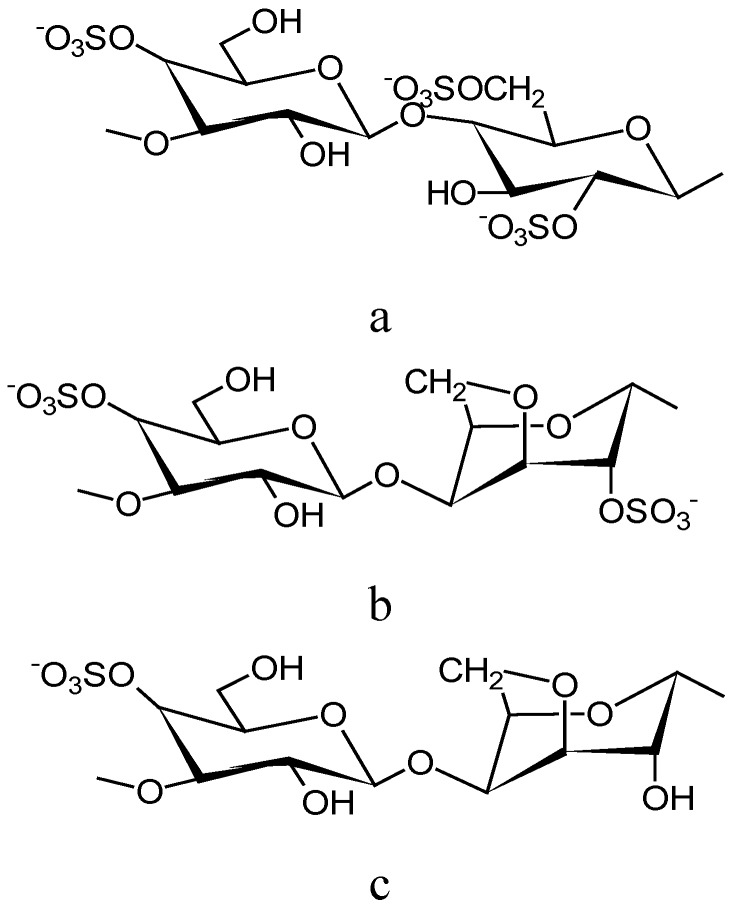
Chemical structure of a) λ-carrageenan, b) ι-carrageenan and c) κ-carrageenan.

Carrageenans is the generic name for a family of high molecular weight sulphated polysaccharides obtained from certain species of red seaweeds belonging to the class Rhodophyceae, especially *Chondrus crispus*, *Euchema spp, Gigartina stellata* and *Iridaea spp* [52,53]. Carrageenan extracted from seaweed is not assimilated by the human body and provides only bulk but no nutrition. There are three basic types of carrageenan (chemical structures are shown in Figure 5): kappa (κ), iota (ι) and lambda (λ) [16]. The λ-type carrageenan results in viscous solutions but is non-gelling, while the κ-type carrageenan forms a brittle gel. The ι-type carrageenan produces elastic gels [47,52].

A study where the compaction ability of two κ-carrageenans (Gelcarin^®^ GP-812 NF and GP-911 NF) and one ι-carrageenan (Gelcarin^®^ GP-379 NF) was investigated showed that these carrageenans are able to form strong compacts with a high elastic recovery. It was finally concluded from the results that the carrageenans investigated were suitable tableting excipients for the manufacturing of controlled-release tablets [54]. In another study, matrices made of ι-carrageenan and λ-carrageenan sustained the release of three different model drugs and showed release profiles that approached zero-order kinetics. It was found that factors such as tablet diameter, drug to carrageenan ratio and ionic strength of the dissolution medium may play a role in the release of drug from these matrices [55].

Hydrogel beads were prepared from a mixture of cross-linked κ-carrageenan with potassium and cross-linked alginate with calcium and they exhibited a smoother surface morphology than that of the one-polysaccharide network beads. The carrageenan parts of the hydrogel pronouncedly enhanced the thermostability of the polymeric network. These beads were introduced as novel carriers for controlled drug delivery systems [56].

## 8. Rosin

**Figure 6:**
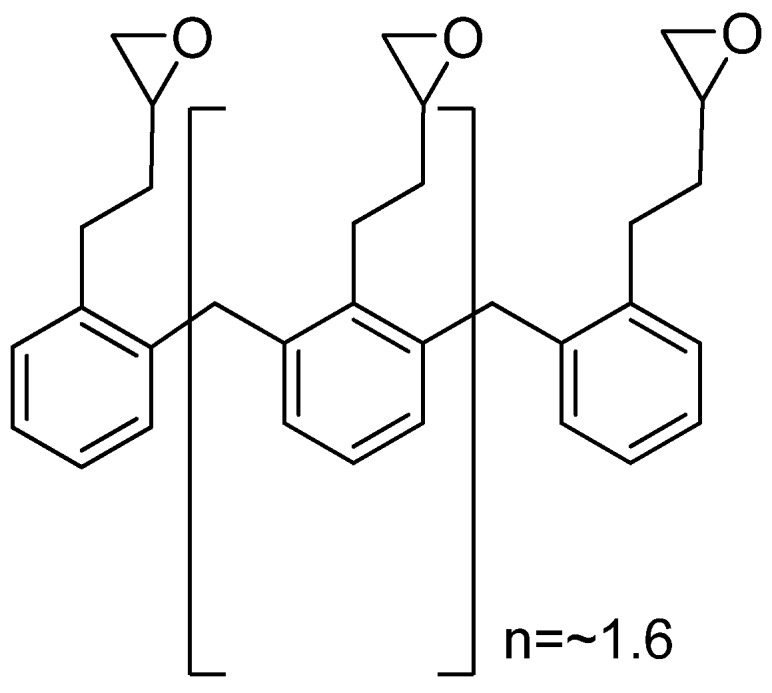
Chemical structure of rosin.

Rosin (chemical structure is shown in Figure 6) is a natural polymer with a low molecular weight of 400 Da obtained from the oleoresin of pine trees, with the principle sources being *Pinus soxburghui, Pinus longifolium* and *Pinus toeda.* Rosin is primarily composed of abietic and pimaric acids and has excellent film-forming properties. Rosin and its derivatives are biopolymers that are increasingly used for their pharmaceutical applications. In the pharmaceutical context it has been investigated for microencapsulation, film-forming and coating properties, matrix materials in tablets for sustained and controlled release [3,57,58].

Derivatives of rosin synthesized by a reaction with polyethylene glycol 200 and maleic anhydride proofed suitable for sustaining drug release from matrix tablets and pellets [59]. Polymerised rosin films containing hydrophobic plasticisers showed excellent potential as coating materials for the preparation of sustained release dosage forms [60]. Different studies on the film forming and coating properties of rosin and the glycerol ester of maleic rosin demonstrated their potential to be used as coating materials for pharmaceutical products as well as in sustained-release drug delivery systems. Furthermore, rosin films showed biodegradation and biocompatibility similar to that of poly(lactide-co-glycolide) [3,61]. It was shown that hydrocortisone loaded nanoparticles prepared from rosin could slowly release this model drug, which was dependent on the rosin content. This *in vitro* study demonstrated the potential of rosin for the production of effective nanoparticulate drug delivery systems [62].

## 9. Gums and mucilages

The term ‘gum’ as applied to water-soluble substances refers to natural non-starch polysaccharides and their structurally modified derivatives. Mucilages is a term that was used to describe the slimy aqueous dispersions produced by plants, animals and microbes, which consist basically of water-soluble polysaccharides including starches and modified starches [63]. Gums and mucilages are used in many pharmaceutical applications such as emulsifyers, suspending agents, binders and disintegrants as well as sustaining agents in tablets and as gelling agents [64].

### 9.1. Guar gum

**Figure 7:**
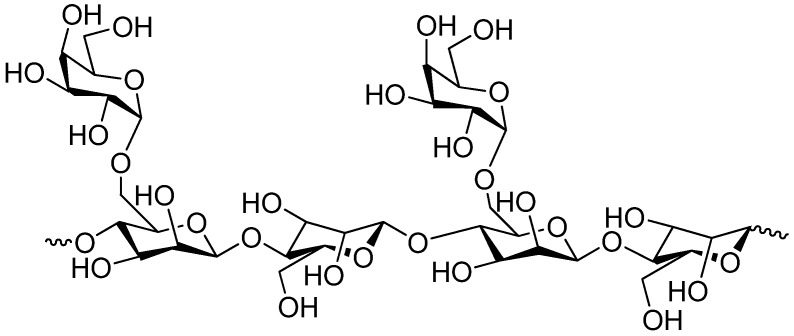
Chemical structure of guar gum.

Guar gum (chemical structure is shown in Figure 7) is also called guaran, clusterbean, Calcutta lucern, Gum cyamposis, Cyamopsis gum, Guarina, Glucotard and Guyarem [65]. Guar gum is a galactomannan, which occurs as a storage polysaccharide in the seed endosperm of plants in the Fabaceae family. Galactomannans are linear polysaccharides consisting of (1→4)-diequatorially linked β-d-mannose monomers, some of which are linked to single sugar side-chains of α-d-galactose attached [66]. Guar gum has a backbone composed of β-1,4 linked- d-mannopyranoses to which, on average, every alternate mannose an α-d-galactose is linked 1→6 [53]. The FDA has affirmed guar gum as generally safe [47]. 

Guar gum has recently been highlighted as an inexpensive and flexible carrier for oral extended release drug delivery [2]. Guar gum is particularly useful for colon delivery because it can be degraded by specific enzymes in this region of the gastrointestinal tract. The gum protects the drug while in the stomach and small intestine environment and delivers the drug to the colon where it undergoes assimilation by specific microorganisms or degradation by the enzymes excreted by these microorganisms. As a hydrogel, guar gum was not found to be highly suitable for controlled release of water-soluble drugs because of their relatively fast delivery, but is useful for poorly water-soluble drugs. It is also used as thickener for lotions and creams, as a tablet binder and as an emulsion stabilizer [47,53]. Guar gum on its own showed high potential to serve as a carrier for oral controlled release matrix systems. In addition, it was found that inclusion of excipients can be used as a tool to modulate drug release from these matrix systems [67].

### 9.2. Locust bean gum

**Figure 8:**
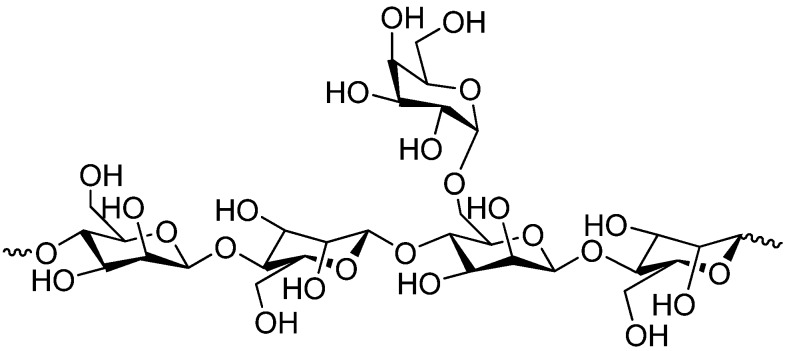
Chemical structure of locust bean gum.

Locust bean gum (chemical structure is shown in Figure 8) also known as Carob bean gum is derived from the seeds of the leguminous plant *Ceratonia siliqua* Linn. This gum is widely cultivated in the Mediterranean region and to a smaller extent also in California. The brown pods or beans of the locust bean tree are processed by milling the endosperms to form locust bean gum and it is therefore not an extract of the native plant but a flour. Locust bean gum consists mainly of a neutral galactomannan polymer made up of 1,4-linked d-mannopyranosyl units and every fourth or fifth chain unit is substituted on C6 with a d-galactopyranosyl unit. The ratio of d-galactose to d-manose differs and this is believed to be due to the varying origins of the gum materials and growth conditions of the plant during production. Locust bean gum is a neutral polymer and its viscosity and solubility are therefore little affected by pH changes within the range of 3-11 [68]. 

Locust bean gum was used to produce matrix tablets with and without the cross-linker, glutaraldehyde, that showed similar drug release profiles for different model drugs as guar gum and scleroglucan [53]. In another study, sustained release of diclofenac sodium could be obtained for mini-matrix systems made from locust bean gum [69]. A commercially available tablet system (TIMERx^®^) developed by Penwest Pharmaceuticals Company consisting of locust bean gum and xanthan gum showed both *in vitro* and *in vivo* controlled release potential [70].

### 9.3. Gum arabic

Gum arabic is a natural polysaccharide which is obtained from the exudates of *Acacia* trees [71]. Structurally, gum arabic is a branched molecule with the main chain consisting of 1,3-linked β-d galactopyranosyl units with other carbohydrates such as arabinose, glucuronic acid and rhamnose also present. Gum arabic was successfully used as a matrix microencapsulating agent for the enzyme, endoglucanase, which proofed to give slow release of the encapsulated enzyme and in addition increased its stability [72]. Gum arabic was used as an osmotic suspending and expanding agent to prepare a monolithic osmotic tablet system. The optimum system delivered the water-insoluble drug, naproxen, at a rate of approximately zero order for up to 12 hours at a pH of 6.8 [73].

### 9.4. Psyllium

Psyllium mucilage is obtained from the seed coat of *Plantago ovata* by milling the outer layer of the seeds. It has been evaluated for its tablet binding properties [74], but also to form hydrogels through radiation-induced cross-linking for controlled release of 5-fluorouracil as model drug [75]. Psyllium and methacrylamide based hydrogels were prepared by using *N*,*N*’-methylenebisacrylamide as cross-linker, which were then loaded with insulin. These cross-linked hydrogels showed controlled release of the active ingredient by means of non-Fickian diffusion of the drug through polymer chain relaxation during swelling [76]. Psyllium husk was used in combination with other excipients such as hydroxypropyl methylcellulose to prepare novel sustained release, swellable and bioadhesive gastroretentive drug delivery systems for ofloxacin [77]. 

### 9.5. Starch

**Figure 9:**
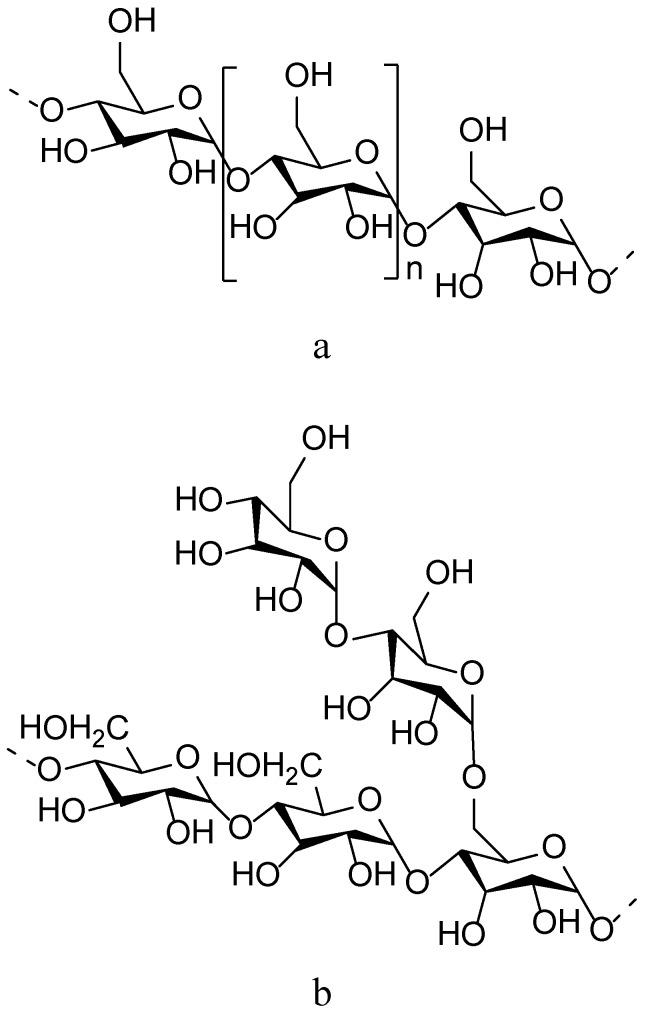
Chemical structure of starch, with a) amylose and b) amylopectin.

Starch is a storage carbohydrate consisting of glucose monomers in plants such as cereals, root vegetables and legumes. It is comprised of two polymers (chemical structures are shown in Figure 9), namely amylose (a non-branching helical polymer consisting of α-1,4 linked d-glucose monomers) and amylopectin (a highly branched polymer consisting of both α-1,4 and α-1,6 linked d-glucose monomers). Most common cereal starches contain 15-30% amylose, but discovery of a recessive gene responsible for production of starch enriched with amylose led to the use of a genetically modified crop for the production of different starches for specific purposes. This has led to the creation of corn producing high amylose starch with a content of 50%, 70% and even up to 90% amylose. Distinct X-ray diffraction patterns are obtained from the crystalline form of starch in granules to distinguish between different types. Type A crystalline structure is characteristic of cereals such as rice wheat and maize, which is favoured by amylopectin with short lateral chains and branching points close to each other. Type B is typical of starch from potato and banana and is favoured by long side chains as well as distant branching points. Type C is found in legumes and is a mixture of the A- and B-type crystalline structures. Starch can also adopt a V-type crystalline structure (derived from the German word ‘Verkleisterung’ meaning ‘gelatinised starch’), which is common for modified starch but is also observed in the endosperm of some native starch granules [78,79]. 

Pregelatinised starch is modified starch by chemical and/or mechanical means to rupture its granules, which results in starch with enhanced flow properties that is direct compressible. Partially pregelatinised grades are commercially available where pregelatinised starch is mixed with free amylase, free amylopectin and unmodified starch in specified ratios. Pharmaceutical grade pregelatinised starch does not contain chemical additives (e.g. salts or bases as gelatinised aids) and is produced by heating and subsequent drying [19]. Starch retrogradation refers to aggregation and crystallisation of starch chains (or precipitation) in gels to form a rigid tri-dimensional network with increased stiffness of the sample [79].

Native starch may not be suitable in controlled release drug delivery systems due to substantial swelling and rapid enzymatic degradation resulting in too fast release of many drugs. This has led to the use of derivatives of starch that are more resistant to enzymatic degradation as well as cross-linking and formation of co-polymers. Starch acetate prepared by acetyl esterification has shown retarded enzymatic degradation with the potential to be used as a colon-targeted drug delivery carrier [80]. Cross-linked high amylose starches have been developed as suitable excipients for controlled release solid oral dosage forms with high loading capacities of active ingredient and with the capability of achieving quasi-zero order release for most drugs. Other advantages include absence of erosion, limited swelling and the ability to control the rate of drug release by changing the degree of cross-linking [81]. A starch product obtained by enzymatic degradation of gelatinised potato starch followed by retrogradation, filtration and washing with ethanol was found to be suitable for the manufacturing of directly compressible controlled release matrix systems [82]. High amylose carboxymethyl starch produced by spray drying showed high loading capacity for the soluble drug, acetaminophen, in controlled release direct compressible matrix systems [83].

Amylose has the ability to form films and when mixed with ethyl cellulose (Ethocel^®^), the films are degradable by colonic bacteria but resistant to gastric acid and pancreatic enzymes upon additional thermal treatment. Amylose and Ethocel^®^ in the ratio of 1:4 w/w showed optimum drug release retarding properties in gastric and intestinal fluids. Another potential coating for colonic drug delivery that has been evaluated is organic solvent based amylose-ethylcellulose films. These films were susceptible to digestion by bacterial enzymes in a simulated colonic environment as reviewed by Sinha and Kumria in 2001 [37]. 

### 9.6. Aloe gel

The inner part of the leaves of *Aloe vera* (L.) Burm.f. (*Aloe barbadensis* Miller) consists of the parenchyma tissue that contains the mucilaginous gel [84]. After extraction of the *A. vera* gel from the leaves and a filtration step, the acetone precipitate was directly compressed in matrix systems with diclofenac sodium as model drug. The mucilage produced direct compressible matrix tablets that showed good swelling and sustained release of the model drug [85].

## 9. Conclusions

Although excipients have traditionally been included in formulations as inert substances to mainly make up volume and assist in the manufacturing process, they are increasingly included in dosage forms to fulfil specialised functions for improved drug delivery because many new drugs have unfavourable physicochemical and pharmacokinetic properties. Several polymers from plant origin have been successfully used and others are being investigated as excipients in the design of dosage forms for effective drug delivery. Some polysaccharides obtained from plants such as carrageenan, alginate, konjac glucomannan, gum arabic, guar gum and locust bean gum have shown excellent potential as carrier materials in matrix type controlled release dosage forms such as microparticles, beads, tablets and cross-linked hydrogels. Plant polysaccharides such as pectin, inulin and rosin have been investigated for their film forming properties, while others have been chemically or physically modified such as the formation of cellulose and starch derivatives. These semi-synthetic polymers are extensively used in the formulation of conventional dosage forms and are under investigation for use in novel drug delivery systems. Plants provide an attractive and renewable source not only for active pharmaceutical ingredients but also for materials that can be utilised as excipients in pharmaceutical products for the effective delivery of drugs.
